# Development of a practical tool to measure the impact of publications on the society based on focus group discussions with scientists

**DOI:** 10.1186/1471-2458-11-588

**Published:** 2011-07-25

**Authors:** Thomas Niederkrotenthaler, Thomas E Dorner, Manfred Maier

**Affiliations:** 1 Centre for Public Health, Department of General Practice and Family Medicine, Medical University of Vienna, Waehringerstr. 13a, Vienna, A-1090, Austria; 2 Centre for Public Health, Institute of Social Medicine, Medical University of Vienna, Rooseveltplatz 3, 1090 Vienna, Austria

## Abstract

**Background:**

A 'societal impact factor' that complements the scientific impact factor would contribute to a more comprehensive evaluation of scientific research. In order to develop a practical tool for its assessment, it is important to learn about perceptions of scientists on how to measure a societal impact factor.

**Methods:**

This qualitative study presents the development of a practical tool to measure the societal impact of publications based on 8 focus group discussions with 24 biomedical scientists at the Medical University Vienna between May 2008 and May 2009. Topics focused on (1) features of an ideal tool, (2) criteria that should be considered in the assessment, and (3) the identification of practical pitfalls. In an iterative exercise involving the repeated application of the drafted tool to scientific papers, criteria for the assessment were refined. A small-scale exercise to evaluate the tool in terms of its comprehensibility, relevance and practicability was conducted using questionnaires for 6 external experts in leading positions of public health, and yielded acceptable results.

**Results:**

The tool developed consists of three quantitative dimensions, that is (1) the aim of a publication, (2) the efforts of the authors to translate their research results, and, if translation was accomplished, (3) (a) the size of the area where translation was accomplished (regional, national or international), (b) its status (preliminary versus permanent) and (c) the target group of the translation (individuals, subgroup of population, total population).

**Conclusions:**

Focus group discussions with scientists suggested that the societal impact factor of a publication should consider the effect of the publication in a wide set of non-scientific areas, but also the motivation behind the publication, and efforts by the authors to translate their findings. The proposed tool provides some valuable insights for further research and practical applications in the topic area.

## Background

To date, more than thousand different scales exist which aim at measuring scientific performance. The most commonly used metrics are citation-based [1], with the Institute of Scientific Information (ISI) impact factor representing the most widespread measure. The impact factor has been shown to influence editorial decision making as well as author citation behaviour [1-4]. In a recent poll including 150 Nature readers, 50% of the respondents said that they had shaped their research behaviours on the basis of the metrics used at their university [5,6]. There has been increasing criticism in the impact factor's use as the gold-standard of measuring scientific performance [7]. Specifically, the common practice of using the scientific impact factor for measuring performance of individual researchers has been repeatedly criticised for being inappropriate in the light of what the impact factor actually measures [1,7]. To this criticism, recent research on the relatedness of different scientific impact measures adds that the impact factor does not seem to be situated at the core of the notion of scientific impact but represents a rather specific aspect of scientific impact [7].

The ever-increasing demand for research that makes a practical difference in terms of translated outcomes reflected, among others, in policy and societal health, has given rise to ideas about the necessity to evaluate scientific research not only in terms of the accomplished scientific impact, but also in terms of policy impacts, service impacts or societal impacts [8-16]. In particular, the societal impact has been repeatedly discussed as a relevant aspect to determine the value of a publication for the society [8,9,13,14,17].

Currently, research which focuses on the question how a societal impact of research may be practically assessed is sparse. One of the first approaches to assess wider impacts of medical research, with a specific emphasis on economic payback, is the Buxton and Hanney Payback Framework [18]. In this framework, five main categories of payback are investigated, namely knowledge, research benefits, political and administrative benefits, health sector benefits, and broader economic benefits. A core feature of this model is an investigation of the interfaces between research and its environment. The model has been successfully adapted and applied to many different fields in recent years including but not limited to health economics, health technology, health services research and clinical research fields [18,19]. Another British project investigated several dimensions of impact of public policy projects, epidemiology and infectious diseases. It provides a framework which assists researchers in describing the effects of their work in different impact areas and includes considerations on the societal impact of research [20]. Even more specifically related to the area of societal impact, a Dutch ongoing project has developed an outline for an evaluation methodology as a basis for further development. This outline includes potentially suitable indicators of the societal impact of research [10,11,17]. It was developed based on the input from a wide range of disciplines including health sciences, architecture, law, social and technical sciences as well as philosophy. Another Dutch project focused on the societal output at the level of medical research departments at the Leiden University Medical Center, and defined stakeholder specific indicators of societal impact in the areas of knowledge production, knowledge exchange, knowledge use and earning capacity [21]. A further structured approach to the assessment of indicators that demonstrate research impact beyond citation count and targets primarily at basic medical and clinical research settings was provided by a US project [15].

Studies related to the perceptions and opinions of scientists on the societal impact of their research publications are currently lacking. Because scientists in applied fields such as public health constitute a highly important group of stakeholders in the translation of research into practice, knowledge focusing on their opinions may contribute to the ongoing endeavours of defining the societal impact of scientific reasearch. Current literature shows that health scientists in applied academic settings are not satisfied with the ways of how the impact of their research (which normally means, the scientific impact of peer-reviewed publications) is evaluated [1,2,4,6,8,9,22]. This dissatisfaction is partially due to the lack of acknowledgement of the societal effects of research in the assessment [9,13,14,17,22]. It was, therefore, the aim of a much-noticed process launched at the Medical University of Vienna [8,22] to qualitatively assess perceptions of biomedical scientists in the public health area about how to assess the societal impact of publications and to create a practical tool to calculate a societal impact factor.

## Methods

### Focus group discussions as a method for investigating group opinions

Focus group discussions were used as the primary method for investigating opinions on the societal impact of research publications in the present study. This approach is useful for obtaining information about knowledge, ideas, opinions and attitudes in a community or group, particularly when little preknowledge about an issue is available [23,24]. As compared to individual interviews, group discussions enhance creativity because they allow for interaction among group members. They are therefore appropriate for the identification of problem-based solutions [24]. The interactive component has been discussed to "empower" research participants because they allow participants to become an active part in the process of developing ideas [24]. Focus group discussions, however, also have important downsides. The interaction of group members which may on the one hand enhance creativity, may on the other hand silent individual voices of dissent [24]. Therefore, a well-considered approach is necessary when composing groups, conducting the interviews and interpreting results [23,24].

### Formation of the Societal Impact Factor Task Force

The Centre for Public Health comprises 5 departments dedicated to general practice and family medicine, social medicine, environmental health, epidemiology and medical psychology, respectively. An invitation to join a task force for the development of a tool to assess the societal impact of publications based on focus group discussions was sent by email to all scientific employees of the Centre for Public Health, Medical University Vienna in December 2007 by the moderator Manfred Maier (MM). Twenty-four scientific employees, with representatives from all departments, accepted the invitation and formed part of the task force (for names and affiliations, see acknowledgement section).

### Mixed purposeful sampling strategy

The outlined composition of the Centre is comparable to many other international academic public health institutions, which makes the Centre an appropriate place to study opinions among a purposeful typical case sample of scientists in an academic public health setting [25]. In order to purposefully eliminate highly influential opinions, which may disproportionally influence the content of the discussions, the opinions of the department chairmen were analyzed in an opinion leader group discussion separate from the other participants [23]. The other groups each involved 5 to 7 scientific employees from different departments. Researchers from different departments were mixed to ensure a more heterogeneous sampling of individuals within the relatively homogenous setting of the Centre, which is expected to result in more diverse opinions and the breaking up of hierarchical structures which may influence the contents of the discussions [23,24].

### Conduction of the focus group discussions

Two rounds of 8 focus group discussions in total, each involving 5 to 7 members of the task force, were conducted between May 2008 and May 2009.

The first round of focus group discussions was dedicated to the questions of (1) what an ideal tool to measure the societal impact should be like, and (2) what specific indicators should be considered when quantitatively assessing a societal impact of publications. The focus groups typically started with an outline of the general purpose of the discussion, and some general warm up questions, followed by a clarification of terms used in the discussion when necessary. The moderator (MM) encouraged all participants to actively participate in the discussion. The discussions involved individual statements by the participants and group interaction.

The contents of the discussions were recorded in writing by an assistant and/or the moderator.

The qualitative data for each group discussion was analyzed inductively with qualitative content analysis, and codes were assigned to the themes related to the explored questions occurring in the discussions. Themes that were mentioned repeatedly were then used to construct a preliminary tool, which was a questionnaire for the self-assessment by authors of the societal impact of own publications. To increase precision, the moderator and 2 other task force members (TN, TED) collaborated on the transformation of the qualitative codes into the self-assessment form. The self assessment form was then sent out to the Task Force members who were asked (1) to apply the form to one own selected publication and (2) to review the self-assessments of two or three colleagues. The members were asked to provide feedback on (1) their perceptions on the suitability of the tool, (2) practical experiences in the application of the tool to his or her own publications, and (3) practical experiences in the assessment of publications from other authors in a further round of focus group meetings, following the same approach as in the first round. This feedback was then used for a revision of the self-assessment questionnaire. A code book defining the terms used for the assessment and the variables (codes) used to assess the societal impact was developed based on the contents of the focus group meetings, in addition to an information sheet for reviewers and authors which aimed at supporting authors and reviewers in their tasks within the application process.

### Exercise to evaluate the tool

To perform a first exercise related to the validation of the tool developed, six international external experts with leading roles in academic public health were invited to test the tool and to provide structured comments. TN and TED developed a questionnaire which allowed the experts to rate the comprehensibility, the relevance and the practicability of the variables used in the societal impact factor assessment questionnaire, and the comprehensibility and usefulness of the glossary and of the information to authors and reviewers on 4-degree Likert scales (1: 'comprehensive", 'very relevant", 'very practicable', 'very appropriate', 'very useful', and 4: 'not comprehensive at all', 'not relevant at all", "not practical at all', 'not appropriate at all', 'not useful at all'). Whenever 3 or 4 was ticked, the experts were asked to specify the reasons of their choice. In addition, the questionnaire included open-ended comments related to the tool, e.g. the time needed for the assessment.

This questionnaire, together with three representative publications from different research areas at the Centre for Public Health, the respective self-assessment forms by authors, a short version of the code book to be used as a glossary, and an information sheet for authors and reviewers as a support material was sent to 6 external experts. Publication A used for this test was a national health report on diabetes mellitus type 2 in Austria [26]. In this report epidemiological data on diabetes, risk factors and complications were compiled and analysed, and national prevention strategies and policy implications were made [27]. Publication B dealt with mental health promotion and behavioural medicine. It analysed the impact of media guidelines for reporting on suicides on the quality of suicide reporting and on suicide rates in Austria [28]. Publication C was a survey on the allocation of training posts to applicants for postgraduate medical education in Austria [29]. The experts were selected based on their leading academic roles at departments of Public Health or General Practice (for names and affiliations, see acknowledgement section).

### Ethics statement

The study was conducted in compliance with the Helsinki Declaration. All participants in the focus group discussions are investigators and co-author the paper and the drafted tool. The need to obtain ethical consent for this study was specifically waived by the Institutional Review Board at the Medical University of Vienna, Austria.

## Results

### Focus group discussions

#### Ideal features of a tool to measure the societal impact

A frequently mentioned feature of an ideal tool to measure the societal impact of a publication was good practicability in the sense of quick and easy to use application. One participant argued that easy application would be crucial for successful implementation in an academic setting. Related to this theme, participants also mentioned that only the most relevant aspects should be used for assessment. Another feature which appeared frequently was that the tool should be comprehensive enough not to leave out important aspects of a societal impact. There was a main consensus in the focus groups that relevant aspects may be different for different public health related fields, and that this implies that an appropriate tool should leave room for a wide set of field-specific relevant aspects.

#### Indicators to be considered when quantitatively assessing a societal impact of publications

The themes that emerged here were situated either at the level of the effect of the publication, or at the level of its original aim. Furthermore, intellectual and practical investments by the authors were considered important by several participants. Related to the effect level, a main point raised in all discussions was how to appropriately show the relatedness of a specific translational outcome to a publication. Several participants argued that it should be the task of the authors to outline a causal chain between the specific publication and a specific outcome.

There were a multitude of views about which effects should be counted as a societal impact, which resulted in a broad consensus that an appropriate tool should allow for an individual description of the specific effect related to the publication achieved. In all focus groups, time and space issues related to the accomplished effects were raised. Several participants felt that lasting translations or translations that go beyond the regional level should be considered of greater societal impact than smaller translations. Several participants also mentioned that the number of individuals influenced by the accomplishment should somehow be considered in the assessment.

At the level of the investments by the authors, several participants mentioned that most, if not all translation effects accomplished required some efforts by the authors. However, there were also a few participants who argued that personal efforts would not be necessary for a translation of research results. Many different views were present for the type of effort that should be counted as a means to achieve translation. Some participants argued that basically all activities beyond the publication should be counted as translational effort, others argued that the societal impact should complement the scientific impact and should focus more on aspects not normally represented in scientific work.

Related to the level of the aim of the publication, there was no clear consensus about its role for the assessment of the societal impact. Several participants discussed that not only the act of 'producing new knowledge' may result in a societal impact. Also aims such as the increase of awareness among the readership or the application of existing evidence for a practical purpose was argued to be an aim relevant for translation later on.

### Tool to assess a societal impact factor

Based on the findings in the focus group meetings, the questionnaire developed consisted of three quantitative dimensions of assessment, that is (1) the aim of a publication ((a) gain of knowledge, (b) application of knowledge, and (c) increase in awareness), (2) the specific efforts of the authors to translate their research results into societal action, and, if translation was accomplished, the size of the translation in terms of (3) (a) the size of the geographical area where the translation was accomplished (regional, national, international), (b) its status (preliminary versus permanent) and (c) the target group of the translation (individuals, subgroup of the population, total population). For reasons of simplicity, MM, TN and TD agreed to assign one societal impact factor point to publications which are consistent with any of the outlined aims, one additional point for any efforts taken by the authors; one, two, and three points, respectively, for a regional, national and international translation; one and two points respectively, for a preliminary and permanent translation, and one, two, and three points, respectively, for translations targeting individuals, subgroups of the population or the whole public. It was agreed upon that, at a later point in time, the quantitative categorization would have to be adapted, based, among others, on the distribution of points achieved by a larger amount of studies assessed for a societal impact.

**Figure 1:**
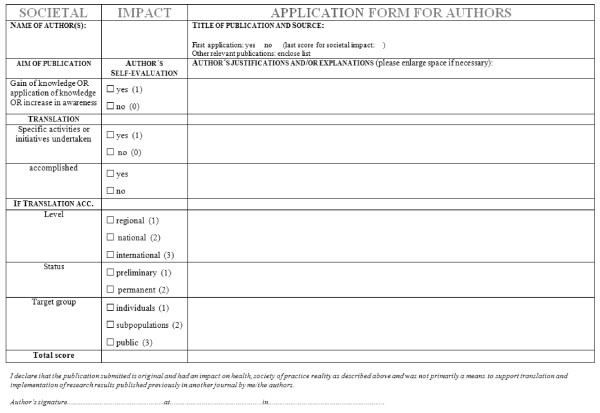
**Application form for a societal impact factor to be used by the applicant for self-assessment**.

**Figure 2:**
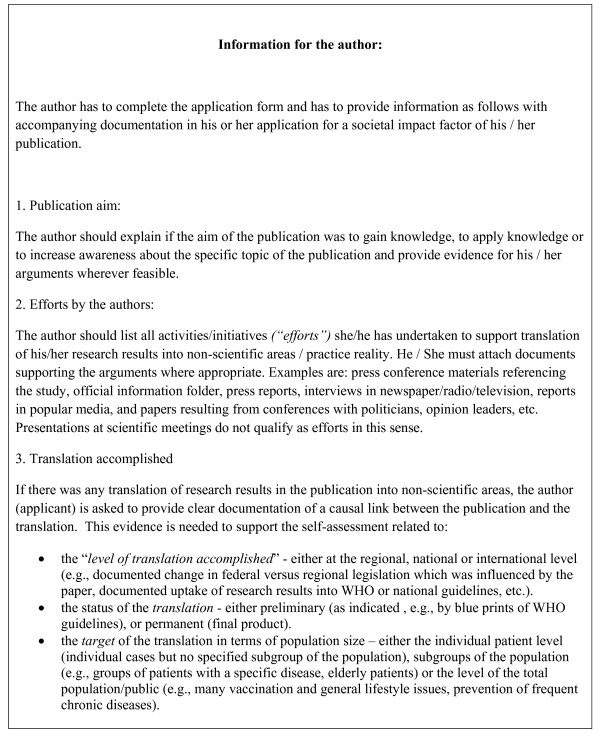
**Information for authors**.

**Figure 3:**
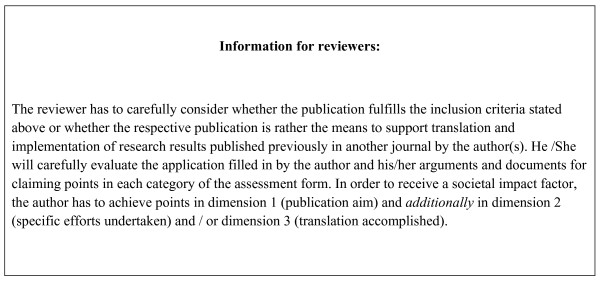
**Information for reviewers**.

The final versions of questionnaire used as the (self-assessment) application form and the assessment form for the reviewer, are given in Figure 1 and additional file 1, respectively. The final version of the glossary is given in table 1. The information to authors and the information to reviewers are shown in Figures 2 and 3, respectively.

**Table 1:** Glossary provided to authors and reviewers

**Societal impact**	
	The use of a research publication in a non-scientific area relevant to the society (e.g. in mass media reports, policies, guidelines).
**Societal impact factor**	Measure to quantify the societal impact.
**Publication**	Scientific publications in journals with or without scientific impact factor and research-based publications in other media.
**Publication aim**	Aim to increase knowledge, to apply knowledge and/or to increase awareness.
**Translation**	Any efforts to make research results applicable to the population (e.g., press conference, mass media report, educative event).
**Accomplishment (of translation)**	An end-point, or more frequently a milestone, at which activities to translate research results receive physical uptake/implementation in an intervention, treatment, policy, or other health-related non-scientific application.
	When assessing the accomplishment of translation of research results into practice reality, it is relevant whether
	• the implementation was realized at the regional, national or international level,
	• the implementation reached a preliminary/pilot or permanent status
	• the implementation targeted at the individual patient case level, at a specified group of the population, or at the total population/society

### Expert evaluation

The comprehensibility, relevance and practicability of the items used for the assessment of the societal impact factor, the glossary as well as the information to reviewers and to authors were rated as acceptable (1 or 2) by all external reviewers. The only exceptions were the relevance and the practicability of the item 'target group", which were rated as 3 by one reviewer. The appropriateness of the complete set of items was rated as being acceptable (1 or 2) by all reviewers.

The time that the reviewers needed for the assessment of the societal impact of one publication ranged from 15 to 180 minutes, with a median minimum time of 20 minutes and a median maximum time of 40 minutes across reviewers.

The rating of publication A by the international experts showed a median of 9 points (ranging from 7 to 10 points). Publication B showed a median of 9.5 points (ranging from 8 to 10 points) and publication C a median of 2 points (ranging from 2 to 6 points), as compared to 10, 10 and 2 points in the self-assessment (application) from the authors, respectively.

## Discussion

The present project examined perceptions of scientists in different fields related to public health about the requirements of a tool to measure the societal impact of publications. Based on the insight gained in the focus groups and based on the repeated applications of the tool developed to scientific studies, it proposes a practical tool to measure the societal impact factor of publications. The tool comprises an assessment of the societal impact of a publication at 3 dimensions: (1) the aim of the publication, (2) the efforts of the authors to translate their research results into societal action or practical reality, and (3) the accomplishment of the translation in terms of the geographical reach (level), status (preliminary or permanent) and size of the target group of this translation. A first exercise to evaluate the comprehensibility, relevance and practicability of the tool, which have been perceived as crucial features of a suitable tool in the focus groups, yielded encouraging results. The time needed by the reviewers for the assessment of the societal impact varied considerably between the experts and the different publications.

The tool developed provides a novel complementary view to earlier projects by exploring the perceptions of scientists working in the field of public health. Compared to previously proposed assessment models, the results of the focus group discussions yielded an assessment model that was largely research-output oriented and is consequently suitable for post-hoc analysis of translation outcomes but not for prospective assessments of possible future translations of research, which are targeted at in other proposed assessment models [17]. In most cases, the societal impact of a publication is reached after the publication of the related study, and develops with time, often depending on the efforts of the researchers to translate their findings into societal action. The societal impact factor assignable to a publication may consequently increase over time, e.g. due to a transition of the translation accomplished from a temporary to a permanent status, or due to an expansion of the translation to the international level following a translation at the regional level. To take future developments into account, authors who apply for a societal impact factor should explicitly be invited to submit their publications for reassessment in case that there is some progress in the translation status.

One of the main differences between the current and earlier approaches is that the proposed tool does not specify which kinds of translations (e.g., which resulting products, e.g. technical devices, guidelines, patents, etc) are specifically considered as accomplished translation. For example, a recently published model differentiates between biological materials, databases, patents, pharmaceutical products, curriculum guidelines, medical devices and many more, which all can be considered some kind of translation outcome [15]. In the present tool, it is completely left to the applicant to describe what his or her translation accomplishment actually is--the only prerequisites made are that there must be some documentation of a causal link between publication and the described beneficial translation outcome, and that the translation is accomplished in a non-scientific area relevant to the society. Compared to other definitions of societal impact used in the literature, the definition used here is wider than in other impact assessment models. This approach makes the tool easier to handle and applicable to different situations, research areas and practice fields. The reasoning behind the exclusion of impact in other scientific areas (as for example indicated by the number of citations of the paper in scientific journals) from the societal impact assessment in this model was based on earlier suggestions that scientific citation-based assessments and societal impact assessments should be synergistic and complementary [21].

Several adaptations and specifications to the developed instrument were made based on the feedback from the external experts. Importantly, the aim of the publication was deemed to be a necessary but - alone - not a sufficient criterion for achieving a societal impact. Additional points in either dimension 2 (efforts undertaken) or dimension 3 (accomplished translation) were deemed necessary to achieve a societal impact factor. A further important specification was made for the 'status of the translation' in dimension 3. Here, it was deemed most suitable not to define a minimum duration of 'permanence' in the light of the ever-changing state-of-the-art practices in biomedical fields. Rather, a translation should be considered permanent if it is part of the official state of the art practice in its subject area at a specific point in time.

The third aspect in dimension (3), that is, (c) 'target group' (1 point: individuals, 2 points: subpopulation, and 3 points: the public) required further specification of the terms used for the categories subpopulation and individuals. In the present form of the instrument, the category 'subpopulation" should be used for any translations targeting directly a definable group of people (e.g., migrants; mentally ill people, depressed patients), whereas the category 'individuals" is for translations affecting individual cases of patients which cannot be described as a coherent group. The latter category may, e.g., be appropriate from the translation of a published case study.

### Strengths and limitations

A strength of the present research is its focus on the perceptions of scientists related to the societal impact of health-related publications, with subsequent translation of the gained insights into a practical tool to assess the societal impact of publications. The focus group interviews provided a relatively quick method to explore opinions in an interactive context and to develop creative problem-based solutions [24]. The repeated applications of the drafted tool to scientific publications and the expert feedback resulted in the identification of several practical issues and subsequent adaptations and refinements that are useful for further research activities and practical applications.

The present study also has several limitations. The applied methodology of using focus group meetings has been criticised in the literature for not taking account of hierarchical structures in groups which influence the contents produced by the groups [30]. In spite of the fact that opinion leaders who may have a high influence on the other participants were purposefully sampled into one group and the moderator (MM) encouraged all participants to take equal part in the process, some related bias in the outcome cannot be ruled out.

Furthermore, the tool developed can be used as an informative practical exercise, but it cannot be considered to be ready for routine implementation. Adaptations and changes will be necessary before it can be used in various applied research fields, and it may turn out to be inadequate for specific fields that were not considered in the present study. Also, the quantitative point assignments to the different categories of the included items are preliminary at the present stage. A categorization into "low impact", "medium impact" and "high impact" is planned for the future when more assessments based on the tool become available. A quantitative assessment seems required to be able to implement the tool in academic settings that are currently (nearly) exclusively considering quick and easy-to-use quantitative assessments of research outputs [5,8].

### Implications for future research

Focus group discussions seemed particularly suitable for the present study based on the small amount of preknowledge on the opinions and beliefs of researchers on the societal impact of their research publications [24]. Future work may use the ideas generated in this analysis to conduct individual interviews or to use consensus-building methods such as Delphi approaches, which can overcome some of the shortcomings of group discussions [31]. The analyses may be expanded to scientists from other fields than public health sciences. Additionally, other stakeholders who contribute to the formation of the societal impact may be included as participants. The tool developed in the present study acknowledges the contribution of other groups of stakeholders than scientists only indirectly by considering interdiscipinary (often non-scientific) work such as media collaborations and policy development.

## Conclusions

Based on the views of scientists working in different fields of public health, the present study provides a novel exercise in developing a tool to measure the societal impact of research publications. The findings provide ample materials to inform internationally ongoing projects aiming at the development of measures to assess the societal impact of research publications.

We encourage internationally ongoing research and application of the tool developed and ask colleagues to comment on the instrument at http://www.societalimpact.info for improvements.

## Abbreviations

ISI: Institute of Scientific Information; US: United States (of America).

## Competing interests

The authors declare that they have no competing interests.

## Authors' contributions

TN and TED contributed to the development of the design and to first drafts of the manuscripts. They contributed equally to the manuscript. MM was the initiator and coordinator of the task force and contributed to the writing of the manuscript. All authors approved the final version of the manuscript.

## Pre-publication history

The pre-publication history for this paper can be accessed here:

http://www.biomedcentral.com/1471-2458/11/588/prepub

## Supplementary Material

Additional file 1 **Assessment form for a societal impact factor**. The assessment form for a societal impact factor which is to be filled out by the reviewer. Click here for file

## References

[B1] Van Noorden R A profusion of measures Nature 2010 465 864 866 10.1038/465864a 20559362

[B2] The Plos Medicine Editors The impact factor game: It is time to find a better way to assess the scientific literature Plos Med 2006 3 e291 16749869 10.1371/journal.pmed.0030291 PMC1475651

[B3] Todd PA Ladle RJ Citations: poor practices by authors reduce their value Nature 2008 451 244 18202622 10.1038/451244b

[B4] Marcovitch H Editors, publishers, impact factors, and reprint income Plos Med 2010 7 e1000355 10.1371/journal.pmed.1000355 21048987 PMC2964337

[B5] Abbott A Cyranoski D Jones N Maher B Schiermeier Q Van Noorden R Do metrics matter? Nature 2010 465 860 862 10.1038/465860a 20559361

[B6] Brumback RA Impact factor wars: episode V--the empire strikes back J Child Neurol 2009 24 260 262 10.1177/0883073808331366 19258283

[B7] Bollen J Van de Sompel H Hagberg A Chute R A Principal Component Analysis of 39 Scientific Impact Measures PLoS ONE 2009 4 6 e6022 10.1371/journal.pone.0006022 19562078 PMC2699100

[B8] Watts G Beyond the impact factor Brit Med J 2009 338 440 441 10.1136/bmj.b553 19218319

[B9] Maier M Men's health and gender medicine: scientific impact or societal impact? J Mens Health 2006 3 330 331 10.1016/j.jmhg.2006.07.006

[B10] Council for Medical Sciences The societal impact of applied health research: towards a quality assessment system 2002 Amsterdam: KNAW

[B11] Spaapen J Dijstelbloem H Wamelink F Evaluating research in context: a method for comprehensive assessment 2007 The Hague: Consultative Committee of Sector Councils for Research and Development (COS) http://www.eric-project.nl/nwohome.nsf/pages/NWOP_82ZCTD_Eng

[B12] Canadian Health Services Research Foundation Insight and Action: Measuring the impact of research: what do we know? (part I) Insight and Action 2008 46 1 4 http://www.chsrf.ca/publicationsandresources/pastseries/insightandaction/08-10-01/eb43c553-38c6-48c9-980b-ef3061fa1987.aspx

[B13] Smith R Measuring the social impact of research Brit Med J 2001 323 528 10.1136/bmj.323.7312.528 11546684 PMC1121118

[B14] Van Driel ML Maier M De Maeseneer J Measuring the impact of family medicine research: scientific citations or societal impact? Fam Pract 2007 24 401 402 10.1093/fampra/cmm061 17998264

[B15] Sarli CC Dubinsky EK Homes KL Beyond citation analysis: a model for assessment of research impact J Med Libr Assoc 2010 98 17 23 http://becker.wustl.edu/impact/assessment/model.html 10.3163/1536-5050.98.1.008 20098647 PMC2801963

[B16] Jenkins LD Maxwell S Change in conservation efforts Bioscience 2011 61 93

[B17] ERIC Evaluating the societal relevance of academic research: a guide (2010). Online resource http://www.eric-project.nl/nwohome.nsf/pages/NWOP_82ZCTD_Eng

[B18] Buxton MJ Hanney S How can payback from health services research be assessed? Journal of Health Services Research & Policy 1996 1 35 43 10180843

[B19] Health Economics Research Group Payback publications http://www.brunel.ac.uk/about/acad/herg/publications/payback

[B20] Kuruvilla S Mays N Pleasant A Walt G Describing the impact of health research: a research impact framework BMC Health Serv Res 2006 6 134 10.1186/1472-6963-6-134 17049092 PMC1635046

[B21] Mostert SP Ellenbroek SP Meijer I van Ark G Klasen EC Societal output and use of research performed by health research groups Health Res Policy Sys 2010 8 30 10.1186/1478-4505-8-30 PMC2964714 20939915

[B22] Niederkrotenthaler T Dorner T Maier M Measuring impact of research on society Nature 2011 469 34 21209648 10.1038/469034c

[B23] Dahlgren L Emmelin M Winkvist A Qualitative Methodology for International Public Health 2007 Umea: Print och Media

[B24] Kitzinger J Qualitative research: Introducing focus groups BMJ 1995 311 299 302 7633241 10.1136/bmj.311.7000.299 PMC2550365

[B25] Mugo FW Sampling in research http://www.socialresearchmethods.net/tutorial/Mugo/tutorial.htm

[B26] Rieder A Rathmanner T Kiefer I Dorner T Kunze M Österreichischer Diabetesbericht 2004: Daten, Fakten, Strategien 2004 Wien

[B27] Dorner T Rathmanner T Lechleitner M Schlögel R Roden M Lawrence K Schwarz F Kiefer I Kunze M Rieder A Public health aspects of diabetes mellitus--epidemiology, prevention strategies, policy implications: the first Austrian diabetes report Wien Klin Wochenschr 2006 118 513 519 10.1007/s00508-006-0666-2 17009062

[B28] Niederkrotenthaler T Sonneck G Assessing the impact of media guidelines for reporting on suicides in Austria: interrupted time series analysis Aust NZ J Psychiat 2007 41 419 428 10.1080/00048670701266680 17464734

[B29] Spiegel W Haoula D Schneider B Maier M Allocation of training posts to applicants for postgraduate medical education in austria: survey and analysis Acad Med 2004 79 703 710 10.1097/00001888-200407000-00019 15234925

[B30] Graefe A Armstrong JS Comparing face-to-face meetings, nominal groups, Delphi and prediction markets on an estimation risk Int J Forecasting 2011 27 183 195 10.1016/j.ijforecast.2010.05.004

[B31] Okoli C Pawlowski SD The Delphi method as a research tool: an example, design considerations and applications Information & Management 2004 42 15 2921789765

