# Supplementary material for: Development of a practical tool to measure the impact of publications on the society based on focus group discussions with scientists
Source: BMC Public Health. 2011 Jul 25;11:588. doi: 10.1186/1471-2458-11-588 (PMC3162524; doi:10.1186/1471-2458-11-588)
Supplement: Additional file 1 — Assessment form for a societal impact factor. The assessment form for a societal impact factor which is to be filled out by the reviewer. [file 1471-2458-11-588-S1.PDF]

SOCIETAL	IMPACT	ASSESSMENT FORM FOR REVIEWERS
**NAME OF AUTHOR(S):**		**TITLE OF PUBLICATION AND SOURCE:**
**AIM OF PUBLICATION**	**REVIEWER'S RATING**	**REVIEWER´S EXPLANATIONS** (please enlarge space if necessary):
Gain of knowledge OR application of knowledge OR increase in awareness	☐ yes (1)<br>☐ no (0)	
**TRANSLATION**		
Specific activities or initiatives undertaken	☐ yes (1)<br>☐ no (0)	
accomplished	☐ yes<br>☐ no	
**IF TRANSLATION ACC.**		
Level	☐ regional (1)<br>☐ national (2)<br>☐ international (3)	
Status	☐ preliminary (1)<br>☐ permanent (2)	
Target group	☐ individuals (1)<br>☐ subpopulations (2)<br>☐ public (3)	
**Total score**		
